# The AKT inhibitor triciribine in combination with paclitaxel has order-specific efficacy against Zfp217-induced breast cancer chemoresistance

**DOI:** 10.18632/oncotarget.19308

**Published:** 2017-07-17

**Authors:** Christopher D. Suarez, Junmin Wu, Sunil S. Badve, Joseph A. Sparano, William Kaliney, Laurie E. Littlepage

**Affiliations:** ^1^ Department of Chemistry and Biochemistry, University of Notre Dame, Notre Dame, IN 46556, USA; ^2^ Harper Cancer Research Institute, South Bend, IN 46617, USA; ^3^ Indiana University School of Medicine, Indianapolis, IN 46202, USA; ^4^ Montefiore Medical Center, Albert Einstein College of Medicine, Bronx, NY 10461, USA

**Keywords:** Zfp217/ZNF217, triciribine, chemoresistance, patient-derived tumor xenograft, microvessel density

## Abstract

We previously identified the transcription factor ZNF217 (human) / Zfp217 (mouse) as an oncogene and prognostic indicator of reduced survival, increased metastasis, and reduced response to therapy in breast cancer patients. Here we investigated the role of Zfp217 in chemotherapy resistance. Preclinical animal models of Zfp217 overexpression were treated with a combination therapy of the microtubule inhibitor epothilone B, doxorubicin (Adriamycin), and cyclophosphamide (EAC). Tumors overexpressing Zfp217 increased their tumor burden compared to control tumors after treatment and accumulated a mammary gland progenitor cell population (K8^+^K14^+^). To overcome this chemoresistance after ZNF217 overexpression, we treated tumors ± Zfp217 overexpression with paclitaxel and triciribine, a nucleoside analog and AKT inhibitor that kills cells that overexpress ZNF217. Treatment order critically impacted the efficacy of the therapy. Combination treatment of triciribine followed by paclitaxel (TCN→PAC) inhibited tumor burden and increased survival in tumors that overexpressed Zfp217, whereas single agent or combination treatment in the reverse order (PAC→TCN) did not improve response. Analysis of these tumors and patient-derived tumor xenograft tumors treated with the same therapies suggested that Zfp217 overexpression in tumors contributes both to decreased microvessel density and vessel maturity, while TCN→PAC tumors overexpressing Zfp217 showed improved vessel maturity.

## INTRODUCTION

Even if diagnosed with early stage disease, approximately one-third of breast cancer patients will have tumors that develop resistance to available therapies [1]. Unfortunately, the mechanisms used by cancer cells to develop resistance are not well understood, which leaves clinicians faced with decisions about the treatment options to use and the timing of these interventions. The survival of these patients requires identification of both novel predictive biomarkers and therapies used to overcome this resistance, particularly in life-threatening metastatic tumors. For this reason, it is beneficial to identify biomarkers that predict which breast cancer patients are at a higher risk of developing therapeutic resistance. Biomarkers can aid drug design, identify treatment regimens with increased efficacy, and prevent unnecessary treatment.

We previously identified ZNF217 (human)/Zfp217 (mouse) as a candidate therapeutic target with prognostic value for resistance to breast cancer therapies [2, 3]. ZNF217 is a Krüppel-like zinc finger protein amplified and overexpressed in ∼25% of breast cancer patients [4] and is a biomarker of poor prognosis [2]. Indeed, patients with tumors that overexpress ZNF217 have reduced survival, increased metastasis, and increased chemoresistance. Zfp217 is oncogenic when overexpressed in mouse models of breast cancer, and this overexpression promotes chemoresistance [2, 5, 6]. ZNF217 overexpression also promotes paclitaxel and doxorubicin resistance in both cell-based and xenograft models [2, 5, 6]. Recent *in vitro* studies also show that ZNF217 overexpression promotes tamoxifen resistance in mammary epithelial cells in culture [7].

Chemotherapy treatment of breast tumors leads to an increase in the percentage of stem/progenitor cells [8]. Stem/progenitor cells are candidate cells that are resistant to chemotherapy after ZNF217 overexpression. We previously found that Zfp217 overexpression promotes an increase in self-renewal capacity, invasion, and metastasis, as well as expansion of a progenitor cell population during both normal mammary development and breast cancer progression [2]. In addition, stem cells remain in chemoresistant tumors [8].

ZNF217 regulates the expression of ErbB3 [9] and promotes activation of Akt kinase activity [2]. Akt is a serine/threonine protein kinase involved in essential cellular processes including metabolism, cell growth, proliferation, cell cycle progression, and survival [10]. It has also been implicated in the pathogenesis of cancer with all three Akt family members having increased expression and activity in breast cancer [11].

Consistent with the AKT signaling pathway being a potential target after ZNF217 overexpression, our lab identified the Akt inhibitor triciribine as the only drug tested *in vivo* that has shown the potential to decrease tumor burden in a human breast xenograft with high ZNF217 expression [2, 12]. Several Akt inhibitors, including triciribine, have been and continue to be tested in clinical trials to treat solid tumors [13]. Triciribine has been explored as a treatment option in a phase II clinical trial for metastatic breast cancer [14].

Even though cell culture and patient data support a role for ZNF217 in chemoresistance, the equivalent *in vivo* experiments testing for chemoresistance in tumors that overexpress ZNF217 have not been completed. In this study, we first determine if Zfp217 overexpression in breast tumors promotes chemoresistance. We then investigate the efficacy of triciribine and paclitaxel combination therapy on these tumors. We also identify novel mechanisms of response to combined triciribine and paclitaxel treatment that will provide mechanistic insight into the increase in efficacy.

## RESULTS

### Increased expression of Zfp217 promotes chemoresistance *in vivo*

**Figure 1:**
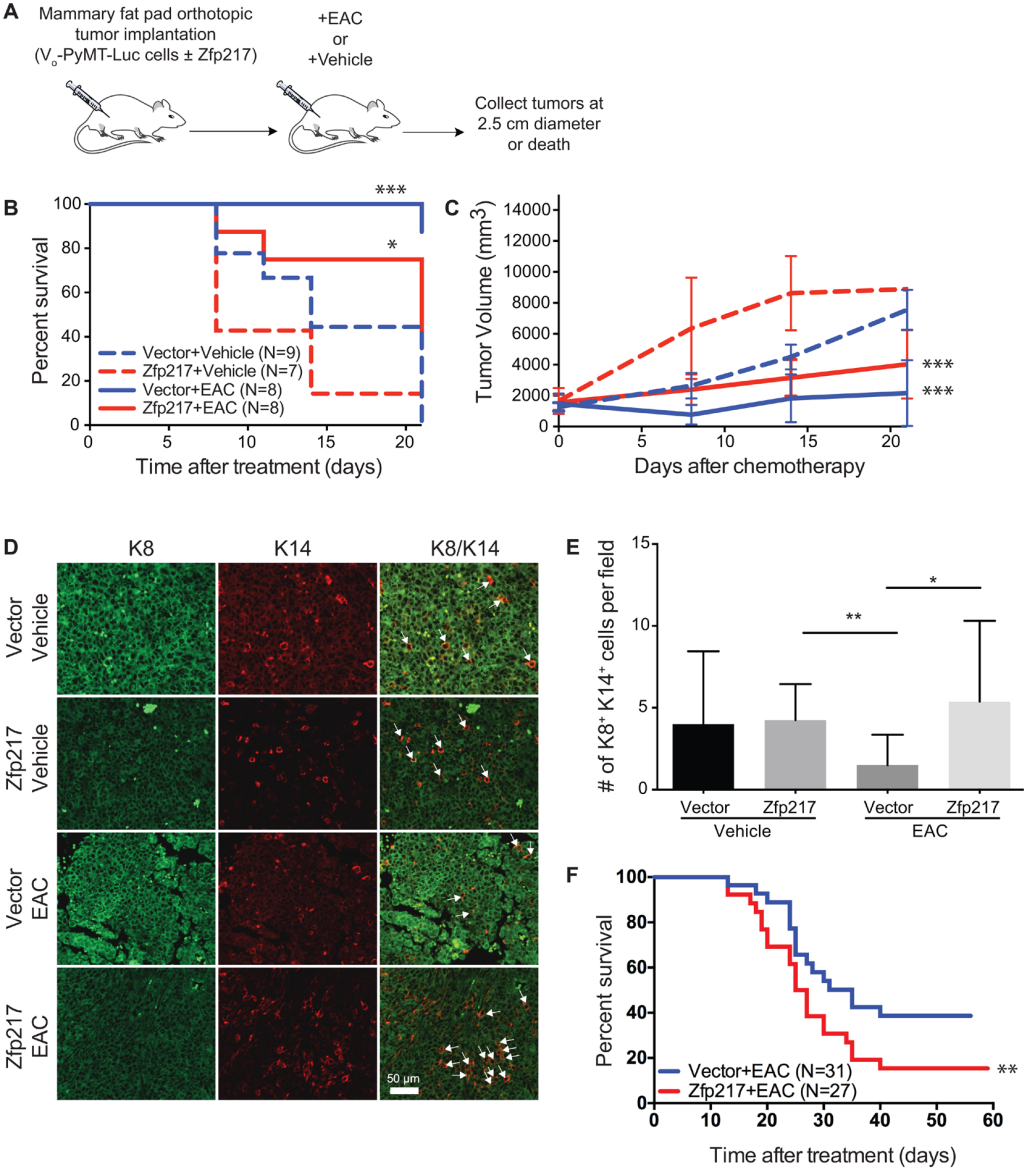
Increased expression of Zfp217 contributes to chemoresistance and an increase in a progenitor cell population **(A)** Experimental overview of orthotopic transplants and combination therapy in orthotopic mammary transplants of Vo-PyMT-Luc cells that constitutively overexpress vector or Zfp217. Mice received a single treatment of epothilone B, doxorubicin (Adriamycin), and cyclophosphamide combination therapy. Tumor tissue was collected at death or at the terminal endpoint when the tumor diameter reached 2.5 cm. **(B)** Kaplan-Meier survival curves of mice bearing orthotopic transplants of Vo-PyMT-Luc cells with constitutive overexpression of vector or Zfp217 after treatment with either EAC or vehicle. Kaplan-Meier survival curves show a significant decrease in median survival between vector and Zfp217 vehicle treated mice compared to mice that received EAC therapy (p=0.0003 and p=0.02 by log-rank test, respectively). The median survival significantly decreases after EAC therapy in mice overexpressing Zfp217 compared to vector (p=0.03 by log-rank test). **(C)** Tumor burden. Tumor volume was compared using linear regression analysis and the slopes of the lines were significantly different between vehicle and EAC treated vector and Zfp217 expressing tumors (p=0.0004 and p=0.0006, respectively). **(D)** K8^+^K14^+^ cells. Tumor sections from FVB mice ± Zfp217 ± EAC were stained for keratin-8 (K8^+^) and keratin-14 (K14^+^) by immunofluorescence staining of tumor sections. Arrowheads point to examples of K8^+^K14^+^ double positive cells. **(E)** Quantification of K8^+^K14^+^ cells. Bar graph showing results of number of K8^+^K14^+^ cells per field. There was a significant increase in the K8^+^K14^+^ double positive cells for Zfp217 tumors treated with EAC compared to tumors expressing vector (p=0.01 by one-way ANOVA with Tukey’s multiple comparisons test). **(F)** Kaplan-Meier survival curves of mice bearing orthotopic transplants of Vo-PyMT-Luc cells with constitutive overexpression of vector or Zfp217 after treatment with EAC. Kaplan-Meier survival curves show a significant decrease in median survival between in Zfp217 + EAC treated mice compared to vector + EAC therapy (p=0.04 by log-rank test). The median survival significantly decreases after EAC therapy in mice overexpressing Zfp217 (26 days, median survival) compared to vector (35 days, median survival).

To determine experimentally if Zfp217 overexpression *in vivo* caused chemotherapy resistance, we generated orthotopic transplants of mouse breast cancer cells ± Zfp217 in syngeneic hosts (Figure 1A). The breast cancer cells used were metastatic mammary epithelial cells derived from MMTV-PyMT [7] ±Zfp217 overexpression by lentiviral infection, as described [2, 15]. Once tumors formed in these animals, cohorts were treated with or without the following three chemotherapies: microtubule inhibitor epothilone B, doxorubicin (Adriamycin), and cyclophosphamide (EAC). Vector control mice treated with EAC had the greatest survival. Consistent with our previous study, vehicle-treated FVB mice with tumors overexpressing Zfp217 had a reduced median survival time of 8 days compared to 14 days for mice expressing vector. Vehicle treated mice with increased expression of Zfp217 also had larger tumor volumes compared to the EAC treated control cohort (Figure 1B) (p=0.0006, linear regression with slope comparison). These data suggest that tumors overexpressing Zfp217 develop resistance to the combination EAC chemotherapy.

### Chemoresistant tumors that overexpress Zfp217 have an expanded progenitor cell population (K8^+^/K14^+^)

To further study this Zfp217 driven chemoresistance, we examined the effects of EAC treatment on a K8^+^K14^+^ progenitor cell population. We previously found that Zfp217 overexpression promotes an increase in a progenitor cell population that expresses markers of luminal (keratin 8, K8) and myoepithelial (keratin 14, K14) cells (K8^+^K14^+^) during breast cancer progression [2]. These K8^+^K14^+^ double positive cells are a bipotent progenitor population capable of forming both luminal and myoepithelial cells [16–20]. We hypothesized that this K8^+^K14^+^ cell population might promote resistance to treatment by expanding a progenitor cell population.

We quantified the K8^+^K14^+^ population in the tumors generated ± Zfp217 overexpression and ± EAC treatment. Interestingly, the chemoresistant EAC treated tumors that overexpressed Zfp217 had significantly increased numbers of K8^+^K14^+^ cells (Figure 1D-1E). Overexpression of Zfp217 significantly increased the percentage of K8^+^K14^+^ cells in mice that received EAC chemotherapy when compared with the vehicle treated mice overexpressing Zfp217 (p=0.01) and with vector-expressing vehicle and EAC treated mice (p=0.001 and p=0.0001, respectively, one-way ANOVA with Tukey’s multiple comparisons) (Figure 1E). In contrast to our previously published results, overexpression of Zfp217 without treatment showed no significant difference in the K8^+^K14^+^ double positive cell population in vehicle treated mice. However, unlike the previous study, the samples in this experiment were collected only when the tumors reached a large size, rather than on the same day. Therefore, treated tumors that overexpress Zfp217 have increased numbers of K8^+^K14^+^ cells compared to vector, and this increase is unlikely due to differences in primary tumor burden and instead demonstrates that the K8^+^K14^+^ progenitor cell population is driven by overexpression of Zfp217.

We also examined the pathology of these tumors and found similar pathologies between the non-necrotic areas of the tumors (Supplementary Figure 1C).

Our data show that Zfp217 overexpression is sufficient to promote accelerated chemoresistance *in vivo* in breast tumors (Figure 1A-1C). Additionally, Zfp217 induced chemoresistance supports the expansion of a K8^+^K14^+^ cell population (Figure 1D-1E).

We next repeated the experiment over a longer time course with more animals to analyze the survival after EAC treatment of animals with breast tumors that overexpress either vector or Zfp217 in a larger cohort (Figure 1F). Similar to our earlier experiment, Zfp217 overexpressing tumors treated with EAC had reduced survival (26 days, median survival) compared to vector overexpressing tumors (35 days, median survival). These results confirm that Zfp217 overexpression accelerates chemoresistance by reducing the survival of these animals.

### Increased expression of Zfp217 contributes to increased lung micrometastases

Increased detection of stem/progenitor cells is prognostic of increased metastasis in patients [21, 22]. After detecting the increase in number of K8^+^K14^+^ cells, we next examined lung metastasis in the same animals. Similar to our previous study, the percentage of mice that developed lung micrometastases was higher after overexpression of Zfp217 (Supplementary Figure 1A-1B). In addition, chemotherapy treatment reduced but did not eliminate lung metastases compared to controls. Despite a small increase in the number of micrometastases in the cohort with tumors overexpressing Zfp217 and treated with vehicle, the quantity of micromets was not significantly different between the EAC treated cohorts (data not shown).

### Dosing order determines tumor-free survival in Zfp217 induced model of chemoresistance

After establishing an *in vivo* model of Zfp217 induced chemoresistance, we sought to identify a novel therapeutic regimen aimed at overcoming Zfp217-induced chemoresistance. We previously identified the nucleoside analog and Akt inhibitor triciribine as a therapeutic with potential to overcome Zfp217 induced chemoresistance [2]. Triciribine currently is in a Phase I-II clinical trial (NCT01697293) in which triciribine is used in combination with the microtubule inhibitor paclitaxel in patients with breast and other cancers. To overcome the Zfp217-induced tumor burden, we examined the efficacy of triciribine as a combination treatment with paclitaxel.

**Figure 2:**
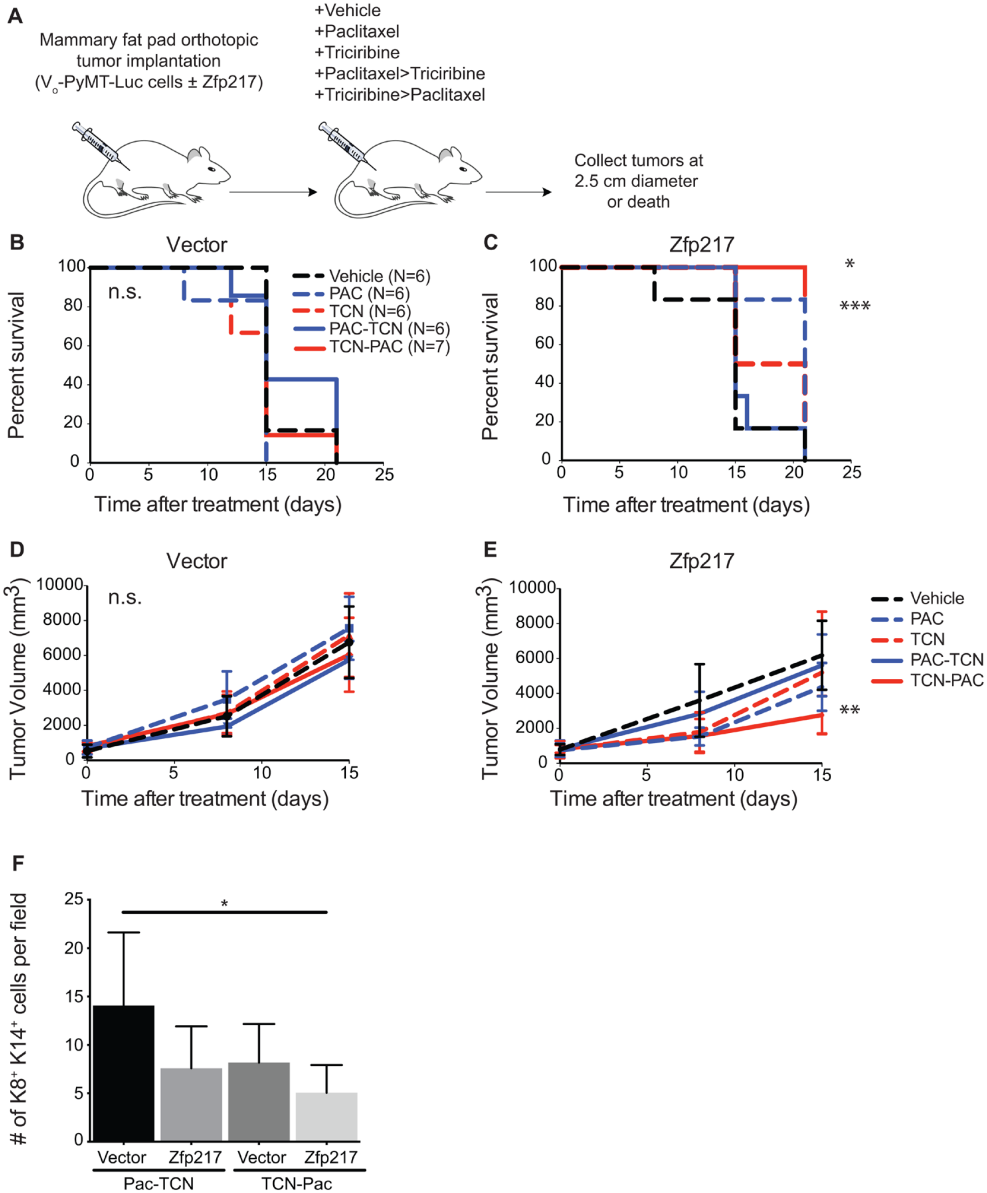
Triciribine-paclitaxel (TCN→PAC) combination therapy increases survival and reduces tumor volume in immunocompetent mice with tumors that overexpress Zfp217 **(A)** Experimental overview. Vo-PyMT-Luc cells constitutively overexpressing vector or Zfp217 were orthotopically injected into recipient FVB mammary glands. Mice received weekly treatments of single or dual agent combination therapy. The dual agent regimen was administered with a delay of approximately 24 hours between delivery of the first and second drug. Tumor tissue was collected at death or at the terminal endpoint, when the tumor diameter reached 2.5 cm. **(B)** Kaplan-Meier curves of recipient FVB mice bearing orthotopically grown Vo-PyMT-Luc cells expressing vector received single or dual agent combination therapy or vehicle. Kaplan-Meier survival curves show no change in median survival between cohorts. **(C)** Kaplan-Meier curves of FVB mice bearing orthotopically grown Vo-PyMT-Luc cells that are constitutively expressing Zfp217 received single or dual agent combination therapy or vehicle. Kaplan-Meier survival curves show a significant decrease in median survival between single agent paclitaxel and dual agent triciribine-paclitaxel compared to vehicle (p=0.0003 and p=0.02 by log-rank test, respectively). **(D)** Tumor burden in vector expressing glands after treatment. Tumor volume was compared using linear regression analysis and the slopes of the lines were not significantly different between vehicle and chemotherapy treated vector expressing tumors. **(E)** Tumor burden in Zfp217 overexpressing glands after treatment. Tumor volume was compared using linear regression analysis and the slopes of the lines were significantly different between vehicle and triciribine-paclitaxel treated Zfp217 expressing tumors (p=0.001). **(F)** Quantification of K8^+^K14^+^ cells. Bar graph showing results of number of K8^+^K14^+^ cells per field for dual agent treated cohorts expressing vector or Zfp217. There was a significant decrease in the K8^+^K14^+^ double positive cells for Zfp217 tumors treated with TCN-Pac compared to vector expressing tumors treated with Pac-TCN (p=0.03, by one-way ANOVA with Tukey’s multiple comparisons test).

To model the design of the Phase II clinical trial (NCT01697293) and to determine if Zfp217 overexpression is predictive of response to triciribine in breast cancer, we tested the efficacy of paclitaxel and triciribine as combination therapy in our preclinical animal models by transplantation of PyMT mammary cancer cells ±Zfp217 overexpression. To determine if the order of drug treatment influenced the efficacy of the treatment, we varied the dosing order within our preclinical study. When tumors formed, we divided the mice into cohorts ±Zfp217 and treated them with either vehicle, single agent, or combination therapies of either paclitaxel followed by triciribine treatment (PAC→TCN) or triciribine followed by paclitaxel treatment (TCN→PAC) for a short time course, at which point some animals began to die from large tumor burden (Figure 2A). We found that the median survival of 15 days and tumor burden from animals with tumors that overexpress vector did not significantly change after any of the treatment combinations [Kaplan-Meier with log-rank (Mantel-Cox) test] (Figure 2B, 2D).

We next compared survival and tumor burden from animals with tumors that overexpress Zfp217 with single agent or combination therapy. Mice receiving the TCN→PAC treatment had the greatest survival (Figure 2C). The mice with tumors overexpressing Zfp217 and treated with TCN→PAC developed significantly reduced tumor burden compared to animals treated with vehicle (p=0.001, linear regression with slope comparison) (Figure 2E). Therefore, TCN→PAC combination therapy exhibits increased efficacy on tumors that overexpress Zfp217, but not vector. In addition, the treatment order matters and significantly influences the survival and tumor burden of mice with increased expression of Zfp217.

In summary, Zfp217 overexpression is predictive of response to triciribine and paclitaxel combination therapy. In addition, Zfp217-overexpressing tumors, but not vector-expressing tumors, were responsive to triciribine when the mice were treated with triciribine prior to paclitaxel treatment, but not when the mice were treated with paclitaxel prior to triciribine treatment.

### Tumors that overexpress Zfp217 and are treated with triciribine and paclitaxel have a reduced progenitor cell population (K8^+^/K14^+^)

We next examined the mechanisms behind the efficacy of the TCN→PAC treatment against tumors that overexpress Zfp217. Because we found the increase in number of K8^+^K14^+^ progenitor cells in resistant tumors that overexpress Zfp217, we reasoned that this population might be eliminated by treatment, which ultimately would reduce tumor burden if these K8^+^K14^+^ cells are the therapy-resistant repopulating cells of the tumor. We quantified the K8^+^K14^+^ population of the TCN→PAC and PAC→TCN tumors (Figure 2F) and found that in mice with tumors overexpressing Zfp217, treatment with TCN→PAC reduced the number of K8^+^K14^+^ cells by 3-fold (p=0.03, one-way ANOVA with Tukey’s multiple comparisons).

**Figure 3:**
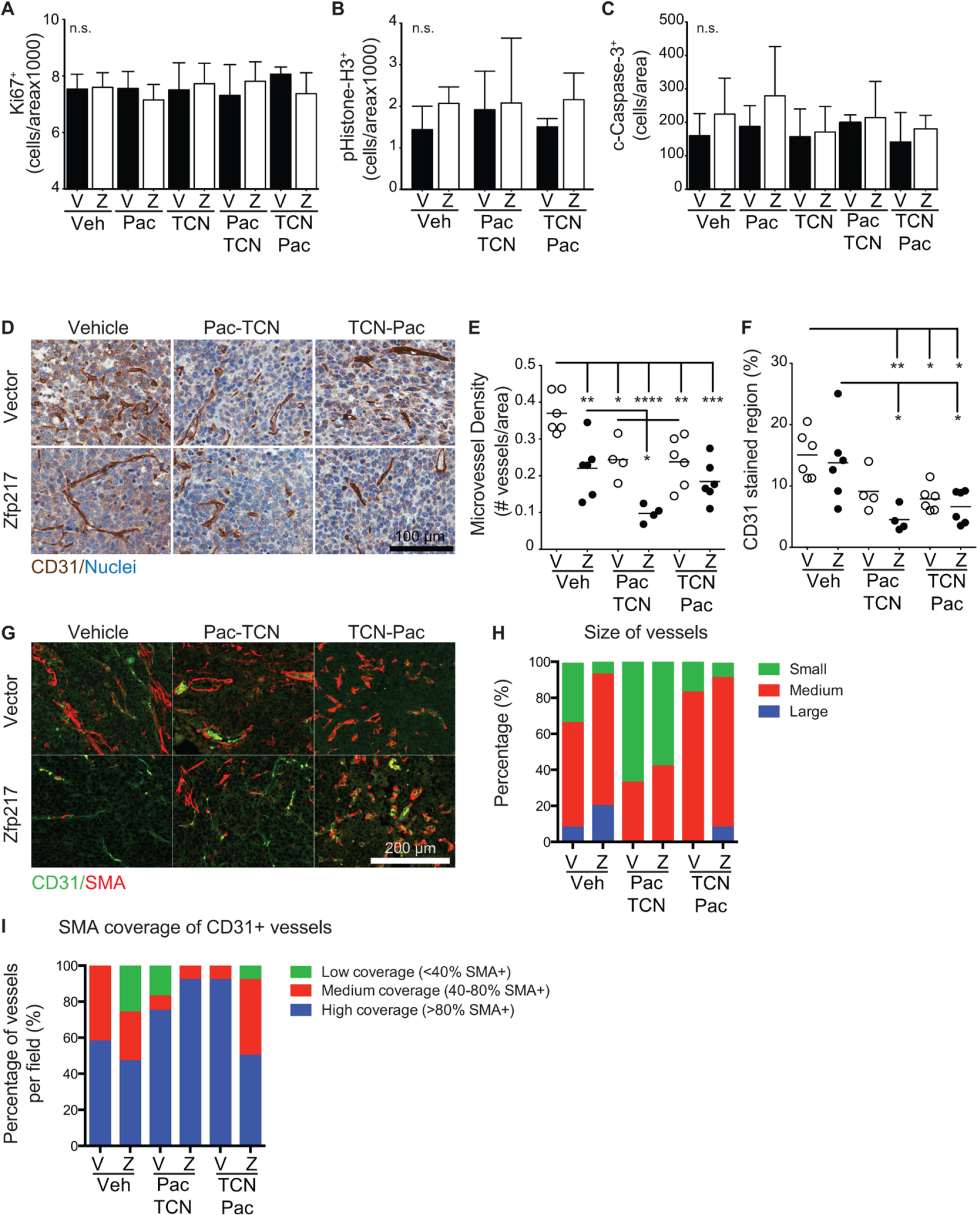
Increased expression of Zfp217 decreases the microvessel density but does not affect proliferation or apoptosis rates **(A-C)** Immunohistochemistry staining and quantification of paraffin embedded sections from vector and Zfp217 expressing tumor tissues. Tissue sections were stained using the ImmPACT DAB detection method and counterstained with hematoxylin. Slides were scanned using Aperio CT, and images were quantified using Aperio software for (A) Ki67, (B) phospho-histone H3, and (C) cleaved caspase-3. Each bar represents the mean ± SD and was analyzed by one-way ANOVA with Tukey’s multiple comparisons. **(D)** CD31^+^ IHC-stained vasculature. Images of IHC stained CD31 positive vessels with hematoxylin counterstained nuclei for each control or treated cohort that expresses either vector or Zfp217. Scale bar=100μm. **(E)** Microvessel density and **(F)** CD31^+^ stained tumor tissue. Scatter plots showing quantified data from Aperio validated microvessel quantification algorithm for (E) CD31^+^ microvessel density (one-way ANOVA with Holm-Sidak’s multiple comparisons) and (F) CD31^+^ stained regions (one-way ANOVA with Holm-Sidak’s multiple comparisons). Bars represent the mean for each cohort (^*^p<0.05, ^**^p<0.01, ^***^p<0.001, ^****^p<0.0001). **(G)** CD31^+^SMA^+^ IF-stained vasculature. Images of tumor sections stained for CD31 and SMA treated with dual agents or vehicle for both vector and Zfp217 expressing tumors. Scale bar=200μm. **(H)** Quantification of size of vessels. Percentage of samples analyzed that had short (small), medium, and long (large) blood vessel size, referring to the vessel length. **(I)** Quantification of SMA coverage of CD31+ blood vessels. Percentage of samples analyzed that had low (<40% SMA+), medium (40-80% SMA+), or high (>80% SMA+) SMA coverage of CD31+ blood vessels.

We also examined the levels of proliferation and apoptosis in tumor tissues ± Zfp217 ± treatment by immunohistochemistry of proliferation markers (both Ki67 and phospho-histone H3) and apoptosis markers (cleaved caspase 3). Levels of neither proliferation nor apoptosis were significantly different across the tumor cohorts, suggesting that there is no difference in proliferation or apoptosis within the tumor region (Figure 3A-3C) (one-way ANOVA with Tukey’s multiple comparisons).

### Zfp217 decreases tumor vasculature response

Since proliferation and apoptosis were not significantly different after treatment or overexpression of Zfp217, we next looked at changes in the microvessel density and maturity as factors that contribute to tumor burden and survival. We quantified the microvessel density after chemotherapy treatment by immunohistochemistry of CD31 (Figure 3D-3F). Interestingly, the tumors that overexpress Zfp217 contained significantly reduced numbers of blood vessels compared to tumors that overexpress vector (p=0.003, one-way ANOVA with Holm-Sidak’s multiple comparisons) (Figure 3E). Treatment with either TCN→PAC or PAC→TCN also decreased the microvessel density in both vector and Zfp217 overexpressing tumors, with particularly significant reduction in the Zfp217 overexpressing tumors. We also found a reduction in CD31 stained area in treated tumors, particularly in the TCN→PAC and PAC→TCN treated tumors that overexpress Zfp217, compared to vehicle (p=0.0096 and p=0.003, respectively, one-way ANOVA with Tukey’s multiple comparisons) (Figure 3F). However, the mean vessel size was not significantly different across cohorts (Supplementary Figure 2) (n.s., one-way ANOVA with Tukey’s multiple comparisons). These results suggest that both Zfp217 overexpression and combination therapy reduce microvessel number and area within the tumors.

To look at the nature of the vessels formed in tumors that overexpress Zfp217, we co-stained the tumors with the endothelial cell marker CD31 and the muscle and pericyte cell marker α-smooth muscle actin (SMA) (Figure 3G). In the vehicle treated samples, the sparse vessels seen in the tumors that overexpressed Zfp217 were longer than the vessels of the vector control tumors (Figure 3H). The tumors with Zfp217 overexpression also had a higher percentage of CD31^+^SMA^-^ vessels than seen in the vector control tumors (Figure 3I), suggesting the vessels in the Zfp217 tumors are either immature or leaky.

We next looked at the tumors treated with PAC→TCN and TCN→PAC. The samples from vector control tumors treated with either drug combination contained predominantly CD31^+^SMA^+^ vessels. The Zfp217 overexpressing tumors from treated animals (PAC→TCN or TCN→PAC) had increased numbers of CD31^+^SMA^+^ vessels, compared to Zfp217 overexpressing tumors treated with vehicle. In addition, PAC→TCN treatment tumors had shorter vessels compared to either the vehicle or TCN→PAC treatment tumors.

The vessels from the TCN→PAC treated animals had increased SMA coverage of vessels compared to vehicle control mice in both vector and Zfp217 expressing tumors. This suggests that TCN→PAC treatment leads to the maturation of the vessels in tumors that overexpress Zfp217.

### Survival status of patient-derived tumor xenografts (PDX) suggests that treatment order matters with triciribine and paclitaxel

**Figure 4:**
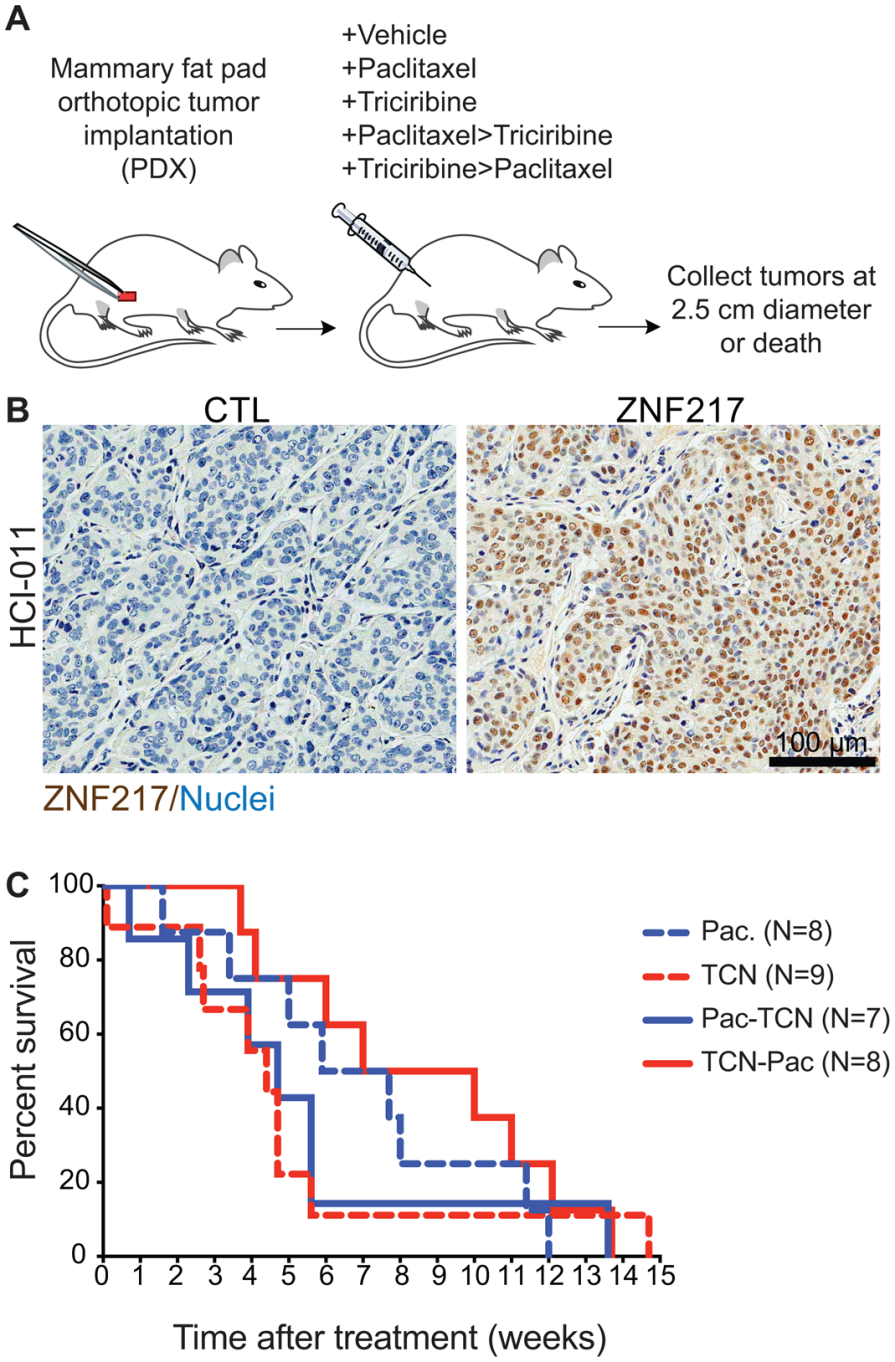
Triciribine-paclitaxel (TCN→PAC) combination therapy has decreased efficacy in tumors from patient-derived tumor xenografts (PDX) grown in immunocompromised mice **(A)** Experimental overview. The timeline depicts the strategy to perform orthotopic mammary transplants of (HCI-011) patient derived tumor xenograft tissues with high expression of ZNF217 using mice with the NOD-SCID background. Mice also received estrogen pellet implants as HCI-011 PDX tissue is estrogen responsive but not dependent. Mice received vehicle, single agent treatment of paclitaxel or triciribine or dual agent paclitaxel-triciribine or triciribine-paclitaxel. Tumor tissue was collected at death or at the terminal endpoint when diameter reached 2.5 cm. **(B)** ZNF217. Images of IHC stained with antibodies raised against ZNF217 show ZNF217^+^ cells with hematoxylin counterstained nuclei. Scale bar=100μm. **(C)** Kaplan-Meier survival curves. NOD-SCID mice bearing orthotopically grown HCI-011 PDX tissue, with high expression of ZNF217, received single or dual agent combination therapy. Kaplan-Meier survival curves show no change in median survival between cohorts.

We used patient-derived tumor xenografts grown in immune compromised NOD SCID mice to determine the efficacy of TCN→PAC compared to PAC→TCN on tumors of human origin that also overexpress ZNF217 (Figure 4A). The PDX tumors used were previously established and characterized for their gene expression levels [23]. The selected model (HCI-011) expressed the highest level of ZNF217 of all of the xenograft tumors tested. Indeed, ZNF217 was expressed at high levels in these tumors (Figure 4B).

Animals with PDX tumors were treated with single agent (PAC or TCN) or combination treatment (PAC→TCN or TCN→PAC) and a Kaplan-Meier survival analysis was completed (Figure 4C). The TCN→PAC animals had a median survival of 9 weeks compared to 5 weeks for PAC→TCN treated animals (n.s. Kaplan-Meier with log-rank (Mantel-Cox) test). However, in contrast to the experiments in immune competent mice, the survival time of TCN→PAC treated mice was not significantly different from PAC treated mice.

**Figure 5:**
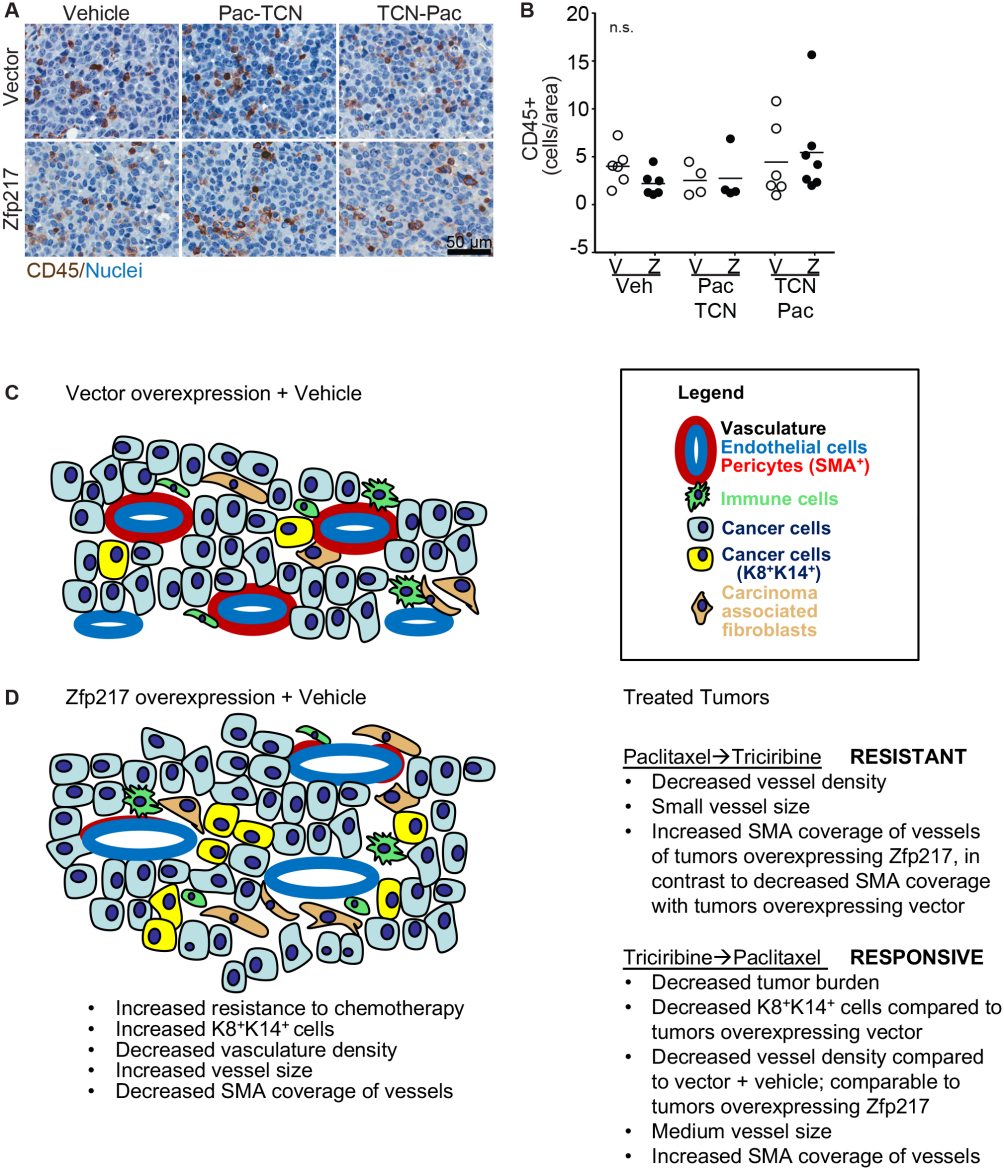
Immunocompetent mice that overexpress Zfp217 were treated with triciribine-paclitaxel and have unaltered CD45^+^ immune cell number **(A)** CD45. Images of IHC stained CD45^+^ cells with hematoxylin counterstained nuclei for each control or dual agent treated cohort. Scale bar=100μm. **(B)** Quantification of CD45^+^ cells. CD45^+^ cells were scored using Aperio software. Each bar represents the mean and analyses were conducted using One-way ANOVA with Tukey’s multiple comparisons. **(C)** Diagram describing results after vector overexpression + vehicle with legend. **(D)** Diagram describing results after ± Zfp217 overexpression + vehicle and after treatment (Paclitaxel→Triciribine, which are resistant to treatment, compared to Triciribine→Paclitaxel, which are responsive to treatment).

We determined if immune cell recruitment contributed to the differences in survival after treatment seen between immune competent and immune compromised animals with tumors that overexpress Zfp217 (same experiment as described in Figure 2). Quantification of CD45^+^ epithelial tumor cells showed no statistical difference between cohorts (n.s., one-way ANOVA with Tukey’s multiple comparisons) (Figure 5A-5B). These data suggest that lymphocyte recruitment is not the primary driver of the differences seen between the immune competent and immune compromised tumor cohorts.

## DISCUSSION

In this study, we found that Zfp217 overexpression promotes chemoresistance associated with an increase in a progenitor cell population. Significantly, we identified a specific dosing regimen of triciribine followed by paclitaxel that can overcome the chemoresistance that results from Zfp217 overexpression. Interestingly, the observed increase in efficacy does not alter proliferation, apoptosis, or CD45^+^ immune cell recruitment, but we do observe changes in the vasculature. To our knowledge, this is the first report of a Zfp217-induced change in the tumor vasculature.

These data suggest that Zfp217 overexpression in the breast tumors promotes vasculature remodeling, marked by reduced numbers of vessels, increased vessel size, and an increased percentage of CD31^+^SMA^-^ vessels compared to vector overexpression (Figure 5C-5D). Interestingly, after TCN→PAC treatment of the Zfp217 overexpressing tumors, not only does the overall survival of the cohort increase for the animals with Zfp217 overexpressing tumors, but also the tumor burden decreases while the vessels of these tumors revert to primarily CD31^+^SMA^+^ vessels. This suggests that the Zfp217 tumors respond to TCN→PAC treatment in part by vascular remodeling and by becoming CD31^+^SMA^+^. This vascular remodeling is consistent with either changes in pericyte coverage or the promotion of arteriogenesis and vasculture maturity and provides a rationale for the improvements seen in the animals treated with TCN→PAC.

The role of ZNF217 in promoting chemoresistance provided the rationale for revisiting the Akt inhibitor triciribine as a candidate for combination therapy in the clinical setting [2]. ZNF217 is required for and promotes increased activation of Akt, which also plays a critical role in promoting chemotherapy resistance in breast cancer cells [2, 5][24]. Several Akt inhibitors have been explored in various stages of clinical development as single agent or combination therapies [13]. Triciribine was the lead drug candidate identified from an *in silico* screen of ∼50,000 drugs specific for cancer cells that have high expression of ZNF217 [2].

In this study, we find that ZNF217 overexpression promotes resistance *in vivo* in response to combination therapies that include not only DNA damaging agents, such as doxorubicin, but also mitotic inhibitors, like the microtubule stabilizer epothilone B. Therefore, we cannot rule out contributions of ZNF217 overexpression to bypassing the G2/M checkpoint, which is the last opportunity for the cell to repair double strand DNA breaks and to prevent DNA damage from being carried into daughter cells. Damaged DNA present during G2/M normally promotes mitotic catastrophe and cell death. Other studies have supported a role for ZNF217 in genomic instability and telomere length. First, ZNF217 overexpression was found to immortalize human mammary epithelial cells (HMECs) through stabilized/increased telomere length and increased telomerase activity [25]. ZNF217 overexpression also increased the numbers of mitotic cells and anaphase bridges, a marker of genomic instability, in cultured cells [26].

Resistance to paclitaxel, which stabilizes microtubules, could also be due to bypassing mitotic checkpoints, which is consistent with the anaphase bridges seen from the samples used in this study (data not shown) and the evidence of genomic instability that we and others have seen after ZNF217 overexpression [25–27]. Indeed, ZNF217 overexpression increases the expression of the mitotic kinase Aurora-A, which we previously found is regulated by ubiquitin-dependent proteolysis and phosphorylation and leads to genomic instability and centrosome amplification [28, 29]. In addition, in cell culture an Aurora inhibitor had synergistic efficacy in combination with paclitaxel [6].

Tumors that overexpressed Zfp217 became resistant to combination chemotherapy treatment and had increased numbers of K8^+^K14^+^, while triciribine→paclitaxel (TCN→PAC) combination therapy led to a decrease in the K8^+^K14^+^ population in the tumors that also overexpressed Zfp217. This suggests that the TCN→PAC treatment reduces the number of cancer stem cells. The increase in K8^+^K14^+^ population is consistent with our findings that ZNF217 overexpression promotes an increase in a mammary stem/progenitor cell population [2]. ZNF217 has been identified as a target gene that is amplified in circulating cancer cells and is part of a signature that predicts response to therapy [30]. In addition, Zfp217 is a required factor for embryonic stem cell (ESC) self-renewal, pluripotency, and somatic cell reprogramming [31]. Zfp217 promotes epigenetic and epitranscriptomic changes that then activate the transcription of pluripotency genes and also regulates the N6-methyladenosine (m^6^A) RNA methylation within the cell [31].

Our study has implications for personalized therapies for patients with breast tumors that overexpress ZNF217. Based on our study, these patients are likely to respond to treatment with triciribine and paclitaxel combination therapy if treated in combination with a gapped schedule of triciribine treatment followed by paclitaxel treatment. Our study suggests that the treatment should be planned with the dose schedule and order in mind.

These pre-clinical data support results observed in breast cancer patients, namely that increased expression of ZNF217 imparts resistance to standard treatments used for breast cancer [2, 32, 33]. This study directly impacted the design of a Phase I-II clinical trial (NCT01697293) testing the efficacy of triciribine in combination with paclitaxel in patients with breast cancer and will impact a future Phase II clinical trial of poor prognosis ER+ metastatic breast cancer patients treated with triciribine. For these and future studies, increased expression of ZNF217 is an ideal prognostic biomarker for triciribine and endocrine therapy clinical trials.

ZNF217 is expressed at the highest levels in breast cancer patients with the worst prognosis. A detailed mechanistic analysis of the contribution of ZNF217 to therapeutic resistance combined with pre-clinical modeling will inform the design of the best treatment plan to kill these cells, thereby reducing mortality in breast cancer patients with aggressive disease.

## MATERIALS AND METHODS

### Animals

Animal experiments were conducted in accordance with the University of Notre Dame Institution Animal Care and Use Committee guidelines (protocol # 15-10-2724). Mice used in this study were maintained under pathogen-free conditions in the University of Notre Dame Freimann Life Sciences animal facility. The immunocompetent mice used in these studies were maintained on FVB/n mice, and the immunocompromised mice were non-obese diabetic severely compromised immune deficient (NOD-SCID) [34]. The patient-derived tumor xenograft model (HCI-011) [23] was used in accordance with IRB approval (protocol # 13-05-1062).

### Tissue culture cell lines and culture conditions

The luciferase expressing murine breast tumor cell line, derived from a polyoma middle T mouse, Vo-PyMT-Luc, was obtained from Conor Lynch [7] and maintained in DMEM-H21 media + 10% FBS. HEK293T cells were purchased from ATCC and maintained in DMEM-H21 media + 10% FBS.

### Plasmids and virus production

The lentiviral plasmid pEIT-Zfp217 and empty vector pEIT, containing the EF1α promoter and tomato fluorescent reporter, was used to generate lentiviral particles to transduce the PyMT cell line [35].

The EF1α promoter-driven tomato fluorescent lentiviral plasmids pEIT and pEIT-Zfp217 expressing a tomato fluorescent reporter gene were used to generate lentiviral particles. Virus was produced as described previously [36]. Briefly, for lentiviral packaging, HEK293T cells were plated in 10 cm tissue culture plates and transfected the following day. Two μg of the transducing vector pLVX, 1.5 μg of packaging vector pCMV-∆R8.2, and 0.5 μg of the VSV envelope vector pCMV-VSVG were co-transfected using calcium phosphate precipitation. The medium was changed the next day, and the cells were cultured for ∼48 hours. Media containing lentiviral particles was collected and filtered through a 0.45 μm filter before storing at -80°C. The lentiviral particles were used at an MOI of 5 to transduce the PyMT-Luc cell line. Virus was titered and equal MOIs (MOI=5) of vector and ZNF217 virus were used to transduce the Vo-PyMT-Luc cells as described previously [2]. After transduction and within one week of expression, the cells were sorted and collected using fluorescence activated cell sorting (FACS) to enrich for the cells expressing the tomato fluorescence protein. This generated the cell lines PyMT-pEIT and PyMT-pEIT-Zfp217.

### Tumor production in animal models

To establish syngeneic tumors, PyMT-pEIT and PyMT-pEIT-Zfp217 cells were injected (1x10^6^ cells in 10 μl) orthotopically into the #4 mammary gland of FVB/n mice. For both experiments, tumors formed by two weeks post-transplant. Tumor measurements were made using electronic digital calipers, and tumor volumes were calculated using the modified tumor ellipsoid formula: Tumor volume =1/2(length x width^2^) [37, 38].

Generation of patient-derived xenografts was approved by the Institutional Review Board and has been described previously [23, 39]. At least six mice per treatment group were used. After tumors reached a minimum of 75 mm^3^ in size, treatment was initiatied. Mice were sacrificed when the tumor diameter reached 2.5 cm in length or width.

### Therapeutics

For the experiment examining syngeneic tumors treated with EAC or vehicle, post-transplant mice were treated after three weeks with epothilone B (intravenous), adriamycin (intraperitoneal), and cyclophosphamide (intraperitoneal) (EAC) combination chemotherapy or DMSO. Nine mice were included in the vector DMSO cohort, seven mice were included in the Zfp217 DMSO cohort, and eight mice were inclued in both the vector and Zfp217 EAC chemotherapy treated cohorts. In another experiment with syngeneic tumors, beginning approximately 2 weeks post-transplant, mice received intraperitoneal injections of paclitaxel followed approximately 24 hours later by triciribine or the reciprocal dosing regimen. Similar to the Phase I-II clinical trial (NCT01697293), administration of triciribine was skipped every 3rd week of the treatment regimen. At least six mice per treatment group were used, and tumors were collected at death or when they reached 2.5 cm in diameter. For the experiment examining overall survival of a larger cohort of syngeneic tumors treated with EAC with a larger cohort, post-transplant mice were treated with EAC combination chemotherapy once the tumors were palpable. Tissues were collected once the tumors reached 2 cm in diameter. This experiment was completed twice, and the results were combined in Figure 1F with the indicated number of mice.

Mice were treated with the indicated drugs at the following concentrations: epothilone B diluted in DMSO (0.6 mg/kg) (LC Labs, Cat#: LC-E-5500), doxorubicin (Adriamycin) diluted in PBS (2 mg/kg) (Sigma-Aldrich, Cat#: D1515), cyclophosphamide diluted in PBS (120 mg/kg) (Sigma-Aldrich, Cat#: C0768), paclitaxel diluted in DMSO (27 mg/kg) (LC Labs, Cat#: P-9600), and API-2 (Triciribine) diluted in DMSO (12 mg/kg) (Tocris, Cat#: 2151).

### Tissue processing

Mammary and lung tissues were collected, fixed overnight in 4% paraformaldehyde, and transferred to 70% ethanol, processed and embedded in paraffin blocks prior to sectioning. Paraffin tissue blocks were sectioned at 4 μm for IHC special stains.

### Antibodies and immunostaining

The following antibodies were used for these studies: CD31 (Abcam 28364) 1:50, CD45 (BD Pharmingen 550539) 1:200, Cleaved caspase-3 (CST9661) 1:200, Keratin-8 (Dev. Stud. Hybridoma Bank-Univ. of Iowa 531826) 1:50, Keratin-14 (Covance PRB-155P) 1:5000, Ki-67 (CST 12202) 1:400, Phospho-Akt (CST 4051) 1:100. All immunohistochemical staining utilized sodium citrate heat-induced antigen retrieval and overnight incubation of primary antibody at 4°C, and mouse antibodies required use of the mouse on mouse kit (Vector Laboratories BMK-2202).

### Image acquisition and analysis

*Analysis of K8/K14:* Fluorescence images were acquired on a Zeiss Axio Observer.Z1 inverted epifluorescence widefield microscope with the Apotome.2 structured illumination slider engaged. Imaris colocalization (x64 version 8.2.0; Imaris) was used to quantitatively assess colocalization of epithelial markers keratin-8 (green channel) and keratin-14 (red channel). Background subtraction was completed using range set to 50 μm. After generating a colocalized image, cells with positive colocalization signal were manually annotated and data was reported as the number of K8^+^K14^+^ double positive cells per high power field. At least three high power fields were analyzed for each section of tissue analyzed.

*Analysis of CD31/SMA:* Images acquired on Zeiss Axio Observer.Z1 using (10X/0.30 M27) EC Plan-Neufluar objective with 1.6X Optovar. Images were acquired using tiles function to capture a 52 image panel using the contour tool to draw a circular region within which the images were captured. The following filters (Ex, Em) and exposure times were used: 43 HE DsRed (545, 572), 440 ms, 38 HE GFP (488, 509), 290 ms, and 49 DAPI (353, 465), 70 ms. The multi-image panel was created using Zeiss Zen pro 2012 software. The full mosaic tile image was opened, and representative areas were zoomed in at 50%.

Images overlayed with Imaris surfaces were acquired using a (40X/0.95 Korr M27) Plan-Apochromat objective with the Apotome.2 structured illumination slider engaged. The following filters (Ex, Em) and exposure times were used: 43 HE DsRed (545, 572), 35 ms, 38 HE GFP (488, 509), 100 ms, and 49 DAPI (353, 465), 12 ms.

Immunohistochemistry images were scanned by an Aperio CT scanner (Aperio Technologies) with a 20X objective. The generated digital images were saved on the eSlide Manager database (ver. 12.0.0.5027). Image analysis for Ki67, p-histone H3, and cleaved caspase-3 was completed using the Aperio Nuclear algorithm with customized macro parameters set to score DAB and hematoxylin chromogen intensities. Image analysis for CD45 was completed using the Aperio Cytoplasmic algorithm with customized macro parameters set to score DAB and hematoxylin chromogen intensities. The raw data generated from the nuclear and cytoplasmic algorithms included percentage of positively stained cells, intensity of the stain, and area of analysis. Image analysis for CD31 was completed using the Aperio CD31 microvessel algorithm with customized macro parameters set to score vessels based on detection of DAB chromogen intensity. The region joining and vessel completion parameters were set to 25 μm and 30 μm, respectively while the dark and light staining thresholds were set to 105 and 165, respectively. The minimum vessel area threshold was set to 50 μm^2^ and the maximum vessel wall thickness was set to 4 μm. The raw data from the microvessel analysis included quantity of vessels, vessel density, vascular area, lumen area, and vessel perimeter. After running the customized macros, a markup image was generated and re-evaluated to confirm accuracy of the algorithms. The data generated was exported from ImageScope annotation files as an Excel spreadsheet and statistical analysis was completed using GraphPad Prism. Figure preparation was completed using the FIJI [40] plugin ScientiFig [41]. Vessel size and SMA coverage were determined manually and scored by two investigators.

### Statistical analysis

Statistical analysis was completed using Prism 6 software (GraphPad Software, Inc.). Unless otherwise stated, more than two cohorts were compared using ANOVA Kruskal-Wallis followed by Dunn’s multiple comparison tests. One exception is for calculating the microvessel density and CD31 stained regions (Figure 3E-3F). In this case, we assumed Gaussian distribution due to the need to have vessels within a particular distance of cells, so we used the Holm-Sidak’s multiple comparison test.

## SUPPLEMENTARY MATERIALS FIGURES


