# Supplementary material for: The AKT inhibitor triciribine in combination with paclitaxel has order-specific efficacy against Zfp217-induced breast cancer chemoresistance
Source: Oncotarget. 2017 Jul 17;8(65):108534–47. doi: 10.18632/oncotarget.19308 (PMC5752462; doi:10.18632/oncotarget.19308)
Supplement: Supplementary file 1 [file oncotarget-08-108534-s001.pdf]

The AKT inhibitor triciribine in combination with paclitaxel has order-specific efficacy against Zfp217-induced breast cancer chemoresistance

SUPPLEMENTARY MATERIALS

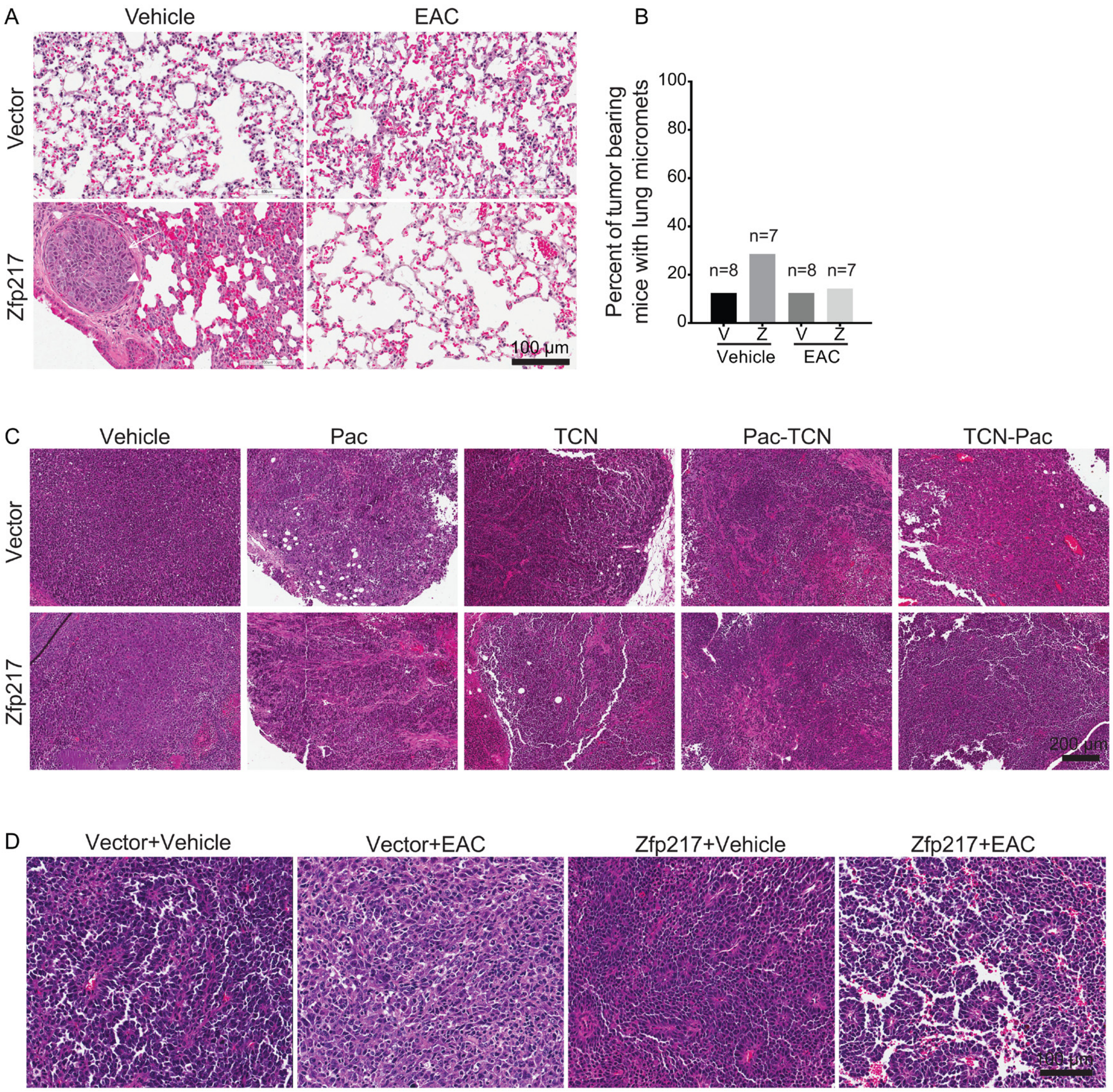

Supplementary Figure 1: (A) Lung H&Es. Representative images of FVB mice ± Zfp217 ± EAC lung tissues that were stained with hematoxylin and eosin. Scale bar=100 µm. **(B)** Quantification of lung micrometastases. The percentage of FVB mice ± Zfp217 ± EAC that developed lung micromets was quantified and there was no significant difference between cohorts. **(C)** Tumor H&Es. Tumor sections from FVB mice ± Zfp217 treated with vehicle, single agent paclitaxel or triciribine or dual agent paclitaxel/triciribine or triciribine/paclitaxel. Tissues were stained with hematoxylin and eosin. Scale bar = 200 µm. **(D)** Tumor H&Es. Tumor sections from FVB mice ± Zfp217 ± EAC were stained with hematoxylin and eosin. Scale bar = 100 µm.

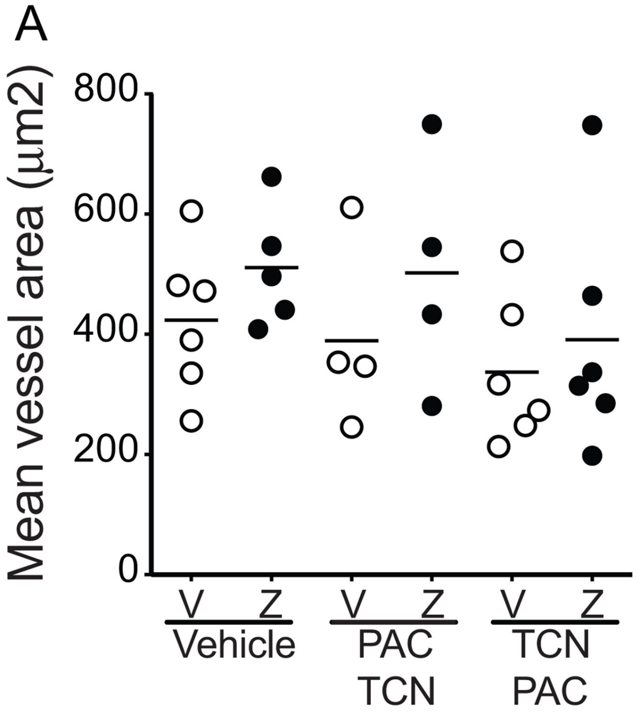

Supplementary Figure 2: (A) Mean vessel area for CD31$^+$ stained tumor tissue. Scatter plots showing quantified data from Aperio validated microvessel quantification algorithm for CD31$^+$ mean vessel area. Bars represent the mean. No significant difference was detected between cohorts.
